# Impact of Exercise and Aging on Rat Urine and Blood Metabolome. An LC-MS Based Metabolomics Longitudinal Study

**DOI:** 10.3390/metabo7010010

**Published:** 2017-02-23

**Authors:** Olga Deda, Helen G. Gika, Ioannis Taitzoglou, Νikolaos Raikos, Georgios Theodoridis

**Affiliations:** 1 School of Chemistry, Aristotle University of Thessaloniki, 54124 Thessaloniki, Greece; oliadmy@gmail.com; 2 School of Medicine, Aristotle University of Thessaloniki, 54124 Thessaloniki, Greece; gkikae@auth.gr (H.G.G.); raikos@med.auth.gr (N.R.); 3 School of Veterinary Medicine, Aristotle University of Thessaloniki, 54124 Thessaloniki, Greece; jotai@vet.auth.gr

**Keywords:** targeted metabolomics, LC-MS, rats, urine, blood, exercise, aging

## Abstract

Aging is an inevitable condition leading to health deterioration and death. Regular physical exercise can moderate the metabolic phenotype changes of aging. However, only a small number of metabolomics-based studies provide data on the effect of exercise along with aging. Here, urine and whole blood samples from Wistar rats were analyzed in a longitudinal study to explore metabolic alterations due to exercise and aging. The study comprised three different programs of exercises, including a life-long protocol which started at the age of 5 months and ended at the age of 21 months. An acute exercise session was also evaluated. Urine and whole blood samples were collected at different time points and were analyzed by LC-MS/MS (Liquid Chromatography–tandem Mass Spectrometry). Based on their metabolic profiles, samples from trained and sedentary rats were differentiated. The impact on the metabolome was found to depend on the length of exercise period with acute exercise also showing significant changes. Metabolic alterations due to aging were equally pronounced in sedentary and trained rats in both urine and blood analyzed samples.

## 1. Introduction

Aging refers to the time-related deterioration of the physiological functions of an organism, which eventually leads to death. It is characterized by a general reduction in cellular function, ultimately affecting the whole body homeostasis. DNA damage, oxidative stress and metabolic dysfunction are common features of aging [1]. 

Metabolomics has been proven a valuable tool for studying the effects of aging on the metabolic phenotype of organisms. Chromatographic tandem mass spectrometry [1,2,3,4,5,6,7,8,9] and NMR spectroscopy [2,8,10,11] have been widely applied to serve the investigation of aging biochemistry. Blood [1,3,7,9], urine samples [2,5,6,8,10,12] and tissues—mostly hepatic tissue samples [1,4]—have been analyzed to study aging. Fecal samples [10,11] have also been used for this purpose. 

Aging metabolomics studies have been published for both humans and rodents [13,14,15]. The findings of these studies could be summarized in the altered metabolism of the associated pathways of amino acids [1,2,4,10], nucleotides [4], glucose [1,2], metabolites of Krebs cycle [2,4,8,10], and lipids [1,2,3,7].

Exercise, and especially physical activity, improves heath and has been proven able to reduce the adverse effect of aging [16]. The physiological [17] and metabolic [18] alterations derived from training are well-established and there are several studies which investigated these through a metabolic profiling approach. In both humans [19,20,21,22,23,24] and rodents [25,26,27,28,29,30,31,32,33,34], metabolomics-based studies decipher changes in amino acids, carbohydrates’ metabolism, Krebs cycle and lipids’ metabolism and other intermediates of biochemical pathways featured as energy providers. These findings on altered metabolism derived from exercise on humans were recently reviewed [35]. For the metabolomics study of exercise in rodent models, GC–MS (Gas Chromatography–Mass Spectrometry) [25,26,27], UPLC–MS/MS (Ultra Performance Liquid Chromatography–tandem Mass Spectrometry) [32,33] and NMR spectroscopy [28,29,30,31,34] have been used to analyze blood [26,28,30], urine [31], and tissue extracts [25,27,29,32,33], mainly from skeletal muscles and hepatic tissue. Recently, a study has provided evidence that rat exercise response imitates human’s response, in blood samples [36].

A small number of studies examines the combined effect of aging and exercise, both of which represent strong metabolome modifiers in rodents [37,38,39,40,41,42]. Of these studies, only one employed metabolomics [42].

In this study, the impact of training and the effect of life-long training against the aging process is explored via their metabolic signatures on rat urine and blood. This is a part of a larger study, where various types of specimens were collected from rats for a long period of time. The results on fecal and cecal tissue metabolome have been previously reported [43]. Here, the effect of exercise and aging in rats is discussed, as a continuation of our previous published work [43], providing a comprehensive picture of the study and supporting previous findings.

## 2. Results and Discussion

### 2.1. Long-Term Exercise Effect on Urine Metabolome

**Figure 1:**
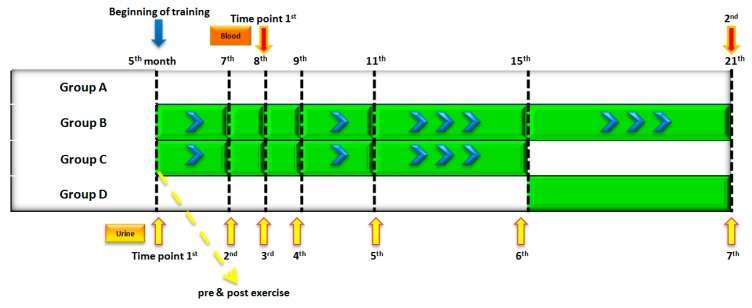
The groups of rats, the training duration and the sample collection time points are illustrated. Urine samples collected at seven sampling time points and one acute training point are marked in yellow. Blood samples collected at two sampling time points are marked in red. *A: sedentary group, B: life-long exercise group, trained from 5 to 21 months of age, C: exercise group trained from 5 to 15 months of age, D: exercise group trained from 15 to 21 months of age*.

In total, 320 urine samples from seven sampling time points were analyzed in order to study the long-term effects of training. By using the applied method, 71 metabolites were detected in the tested urine samples. In Table S1 of the Supplementary Information, the metabolites detected by the applied method are listed. The detected metabolites are mainly amino acids, nucleic acids, carboxylic acids, carbohydrates, hormones, transmitters, vitamins, cofactors, and others. Figure 1 shows an overview of the experiment, indicating the time point of blood and urine samples collection.

**Figure 2:**
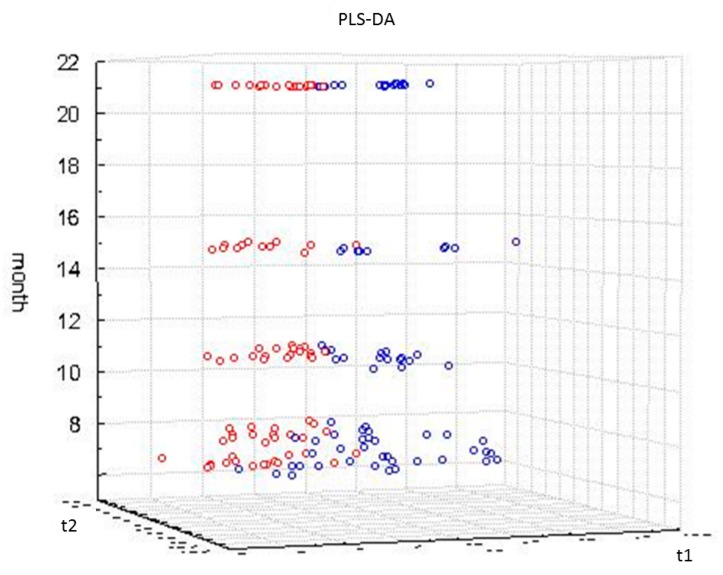
A three-dimensional PLS-DA scores plot of rat urine samples of life-long exercise group B (red colored) and sedentary group A (blue colored) projected in time for five sampling time points, namely 2,3,5,6 and 7 (from down to up), corresponding to 2,3,6,10 and 16 months of training. The Z axis shows the rat age in months while the horizontal axes show t1 vs. t2 and describe the sample variability where the exercise effect is shown.

Looking into the profiling data, it could be seen that the effect of exercise does not seem so pronounced at the first time point. When the following time points were examined, a much clearer effect could be seen. The OPLS-DA model for the data of time point 2, corresponding to two months of exercise, exhibited separation of the exercise group B from the sedentary group A, however, the permutation plot showed that this distinction is not statistically strong. Additionally, univariate statistical analysis by *t*-test failed to give statistically significant alterations for the analyzed metabolites. On the contrary, when the consecutive sampling points were examined, strong trends could be found. At sampling points 3 and 4, exercise could reveal statistically significant differentiators by multivariate and univariate statistical analysis, whereas after six months of training (time point 5), the effect was even more pronounced. The permutation plot and the *p*-value of CV-ANOVA (ANOVA of the Cross-Validated residuals) of the OPLS-DA model indicated a strong differentiation between groups A and B. The OPLS-DA scores plot for the fifth sampling time point can be seen in the Supplementary information, Figure S1. At this point, it should be noted that at sampling time point 3, rats swam with a maximum load in their tails, while at sampling time point 5, rats swam weightlessly. It can be assumed that the longer period of training had a higher impact in urinary metabolic profiles in comparison to the intensity of the exercise. Similar findings were observed in our previous analysis on fecal metabolic profiles of the same study groups [43]. Regarding the time points 6 and 7, corresponding to 10 and 16 months of training respectively, again the effect of exercise was clearly demonstrated. OPLS-DA scores plots for the last sampling time point (seventh) is given in the Supplementary information, Figure S2. In Figure 2, the scores plot for five selected time points after 2, 3, 6, 10 and 16 months of training is given. This shows the clustering of trained rats’ urine samples, apart from the urine samples of sedentary rats. It can be observed that the two groups, A and B, show superior separation after 6 months of training.

Based on univariate and multivariate statistical analysis, the metabolites that were found to differentiate between groups A and B after 6 months swimming are alpha-hydroxyisobutyric acid, 2-hydroxy-3-methylbutyric acid, 3-methylhistidine, acetylcarnitine, arabinose, biotin, cotinine, creatinine, cytidine, glutamine, hypoxanthine, myoinositol, methionine, methylamine, pantothenic acid, threonine, gamma-aminobutyric acid and leucine listed in descending order of VIP (variable importance in projection) value. The AUC (the area under receiver operating characteristic curves) values for the above metabolites were ranged from 0.591 to 0.656.

The same 18 metabolites were also found to have discriminatory value in the last sampling time point (16 months of training) together with four more metabolites, asparagine, *N*-acetylaspartic acid, xylose, sucrose and riboflavin.

**Figure 3:**
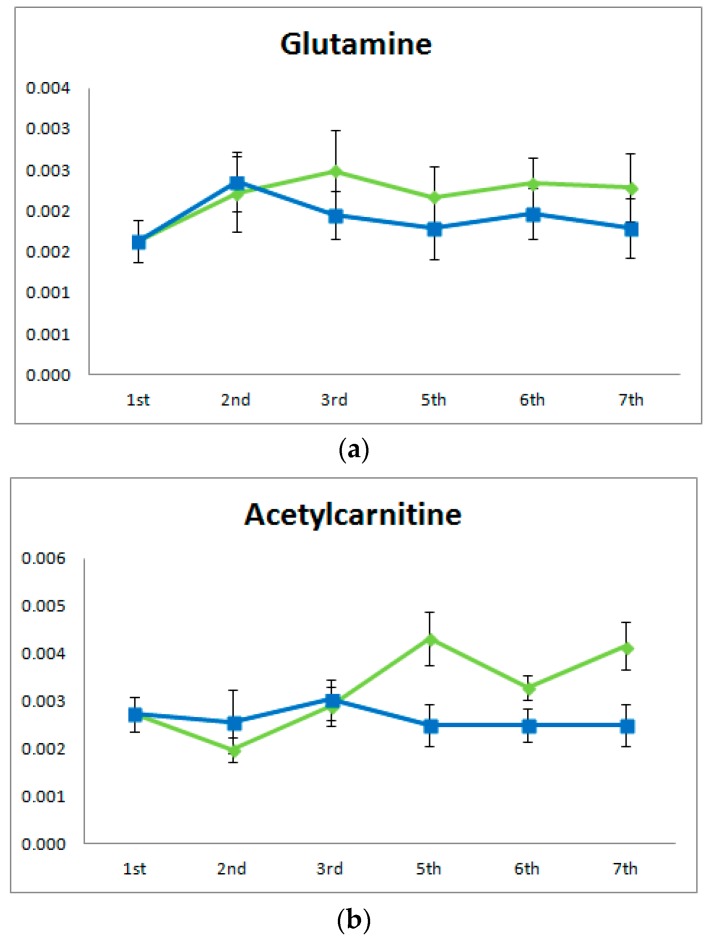
Average of peak areas of rat urine samples of exercise group B (green colored) and sedentary group A (blue colored) at six sampling time points. (**a**) Glutamine; (**b**) Acetylcarnitine.

For better data interpretation, further univariate statistical evaluation was realized on the common compounds that were changed along all sampling time points. In Figure 3, examples of two metabolites which are shown to differentiate with exercise are provided with their trend along aging. It was found that glutamine and acetylcarnitine showed higher response in the samples derived from trained rats in comparison to those derived from sedentary rats, while the opposite happens for creatinine. A descending trend is also observed in other metabolites along aging.

To conclude, exercise-differentiated compounds appear to be related to amino acids’ metabolism. In detail, biochemical pathways of valine-leucine-isoleucine, alanine-aspartic-glutamic acid, cysteine-methionine were most likely affected by swimming. In addition, other compounds, well associated to exercise, such as hypoxanthine and acetylcarnitine were evidenced in potential changes in purines’ catabolism and lipids’ oxidation. Hypoxanthine, along with other purine metabolisms’ derivatives, is a valuable marker of the effectiveness of training [35]. The mild exercise protocol that was adopted, simulated physical activity. Thus, the acceleration of purines’ degradation as precursors of uric acid possibly happened, but not at the same intensity as in vigorous exercise protocols [44]. Furthermore, hypoxanthine, may be altered in the presence of unchanged xanthine and uric acid, caused by the depletion of the enzymatic pathway, of xanthine-oxidase activity or by the inhibition process [45].

Furthermore, when groups B and C, which represent the life-long exercise animals and those which were trained for a long period of their life and then quit (no training for the last six months), are examined, no differences could be detected in their urinary metabolic profiles. This finding suggests that the impact of continuous training has a profound metabolic effect which remains even after a period of 6 months in rats.

Furthermore, no differentiation could be seen when group D (training for a 6 month period in the latter stage of their life) was compared with the group of sedentary rats at time point 7. It seems that rats that have never been trained had similar urine metabolic profiles with rats that were trained only for the last six months, suggesting that the initiation of exercise in older rats was not found to influence the specific set of metabolites monitored by the applied method. It should be noted that the exercise protocol of group B and D in the last 3 months was of lower intensity (i.e., only 2times per week and weightlessly), adapted with animals aging.

### 2.2. Acute Training Effect on Urine Metabolome

To study the acute training effect in urine, samples collected from the same animals belonging to group C at their fifth month of age (Figure 1), right before and after an acute training session, were also analyzed (*n* = 22, *n_i_* = 11 prior, *n_j_* = 11 post).

**Figure 4:**
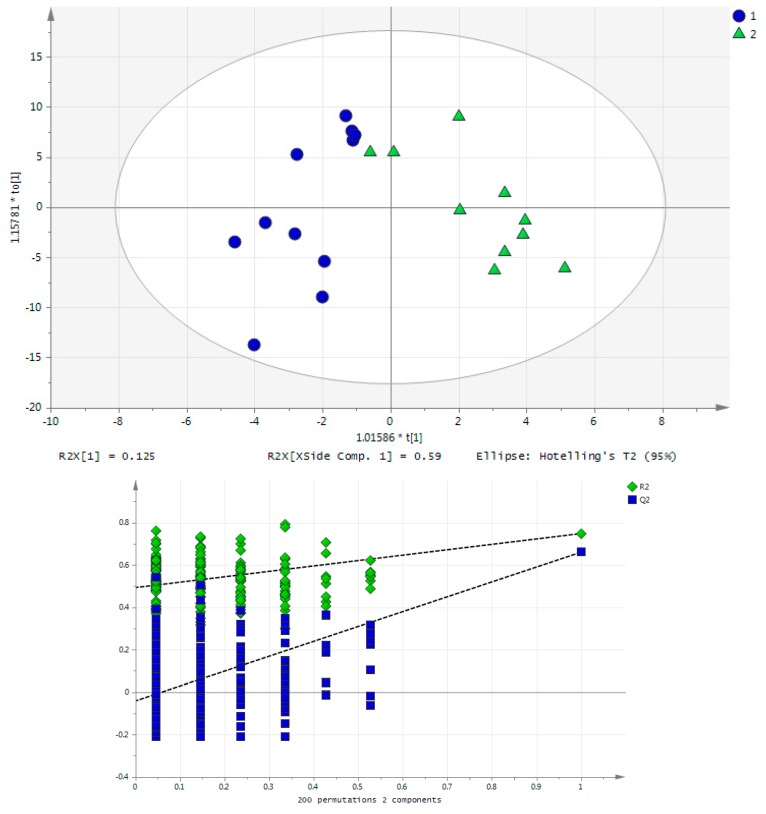
OPLS-DA scores plot of rat urine samples pre- (blue) and post- (green) acute exercise session. The inset permutation plot demonstrates a statistically significantly model.

There was a clear effect of exercise in the urinary metabolites post-training. The differentiation could be described by a PCA model (shown in the Supplementary information, Figure S3), but it is even more clear in the OPLS-DA scores plot of Figure 4, where the inset permutation plot confirms the reliability of the constructed model. In Table 1, VIP scores and *p*-values of the compounds found to be responsible for differentiation between pre- and post-acute exercise groups are presented. The AUC values for the differentiated metabolites were ranged from 0.545 to 0.842.

It can be seen that the significant metabolites are not exactly the same as in long-term exercise. Metabolite alterations derived from acute exercise shared differences from those derived from long-term exercise adaptations. 

Careful examination of the differentiated compounds indicates the biochemical pathways in which these are involved. The metabolic pathway of purines and of arginine-proline, thiamine, nicotinic acid-nicotinamide, appear to be involved in greater scale, following the “pathway analysis” using Metaboanalyst 3.0 [46]. The contribution of the pathways is only a first glance of examining the obtained data. The compounds found to differentiate in pre- and post-exercise rat urine samples, were further examined for each rat individually. It was found that inosine, niacinamide, putrescine and thiamine showed the greatest fold change (%). 

Thiamine plays an important role in metabolism as it is a coenzyme of several key enzymes such as pyruvate, oxoglutarate and branched-chain dehydrogenase. Physical activity can alter the related pathways and thiamine requirements are associated to energy demand [47]. Other post-exercise changes also included increase of nicotinamide due to the conducted enhancement of intracellular respiration in order to reproduce high energy compounds. Tryptophan-related metabolic pathways are well associated with the previous finding and with the increase of insulin release [22,48,49]. 

### 2.3. Impact of Aging 

As our study was longitudinal (over a period of 21 months), changes in the urinary metabolite profiles due to aging are expected to be pronounced. The effect of exercise was studied independently at various sampling points, by comparing animals of the same age. However, when we aimed to investigate the effect of training as a general aspect over aging, all sampling points across the experiment were considered. 

**Figure 5:**
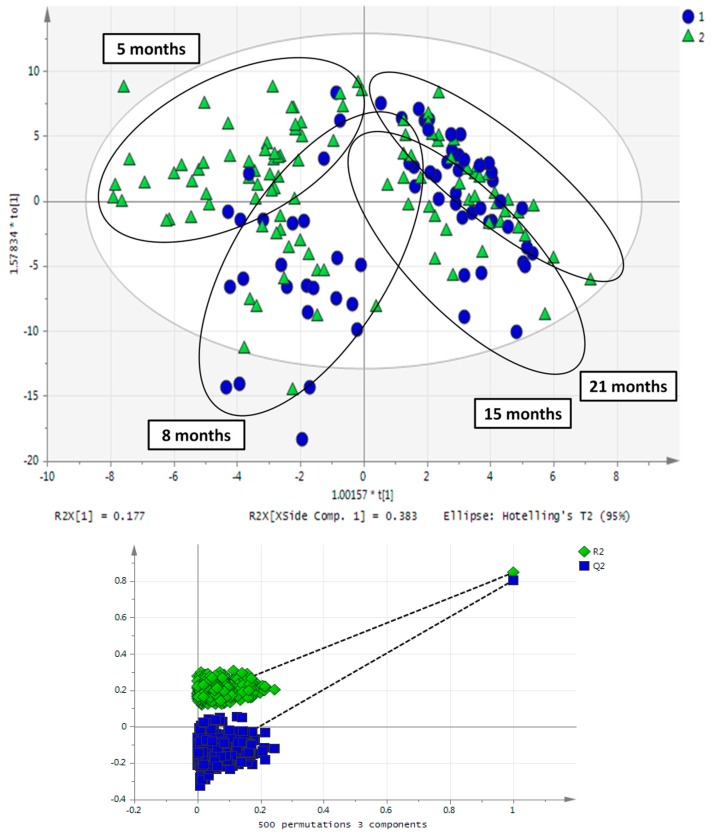
OPLS scores plot of rat urine samples of four sampling time points, corresponding to 5, 8, 15 and 21 months of rat age. Sedentary rats are in blue and exercise in green. It can be seen that the aging factor is distinct while the training rat samples cannot be differentiated from controls when samples from all ages/sampling points are considered for statistical analysis.

In Figure 5, the OPLS scores plot of sedentary and life-long training rats’ urine samples from four sampling time points, corresponding to 5, 8, 15, 21 months of rat age, can be seen. Based on the constructed models, it can be concluded that aging overrules differences due to exercise as these are expressed in the metabolic phenotype described in the first two principal components. Models comparing sedentary and exercise groups at specific sampling time points separated the groups (Figure 2). In contrast, models examining the separation between the two groups along with aging demonstrated lower statistical significance. A similar finding was observed on rat fecal metabolome, where age was found to be the strongest metabolome alteration factor [43].

The comparison of life-long exercise rat (group B) samples between the first and the last sampling time points clearly proved the aging effect on rat urine metabolome (Figure S4). The respective comparison was made for the sedentary rats (group A), providing similar separation (Figure S5). Based on the two PCA models which exhibited similar separation of young and elderly rats’ samples, it can be concluded that the impact of aging in urine metabolome is distinct and that exercise does not seem to invert it. 

In the case of group B, 35 metabolites were found to be responsible for the separation, while for group A, the respective number was 29 (22 in common). These included mainly amino acids, carbohydrates and other intermediate products of metabolism. From the metabolites that were found to be altered due to aging (2-hydroxy-3-methylbutyric acid, arabinose, betaine, choline, cotinine, creatinine, γ-aminobutyric acid, glucose, histamine, myoinositol, kynurenic acid, leucine, methionine, methylamine, valine, pantothenic acid, sarcosine, thiamine, threonine, uridine, xylitol, xylose) ten were also affected by exercise. 

In Table S2, the metabolites affected by both factors (aging and exercise) are provided together with their fold change (%). Τhe fold change of column “aging” was estimated for samples from group A, in the fifth and 21st month of age (sampling time points 1 and 7 respectively); while for column “exercise” the estimation was for life-long exercise and sedentary rats in time point 7. The majority of the metabolites show a decrease with aging while they increase with exercise.

### 2.4. Effect of Training on Blood Metabolome 

In vivo rat blood collection proved challenging. Current practices were tested and different degrees of hemolysis were observed leading to dissimilar blood samples. Thus, whole blood was preferred as a sample to conduct our study. 

Overall 45 metabolites were identified in rat blood samples by the applied method (Table S3). In total, 109 rat whole blood samples from two sampling time points were analyzed. Samples which were collected at the age of 8 and 21 months, corresponding to 3 and 16 months of training respectively, were analyzed and compared. 

It was expected that, due to homeostasis mechanisms, endogenous metabolites alterations by exercise would be less pronounced in blood compared to the effect found in urine. Nevertheless, training had a noticeable effect on the levels of blood metabolites in both time points, demonstrating that whole blood can serve as a reliable type of sample to assess exercise effect, although urine samples are generally preferred for such studies [31].

**Figure 6:**
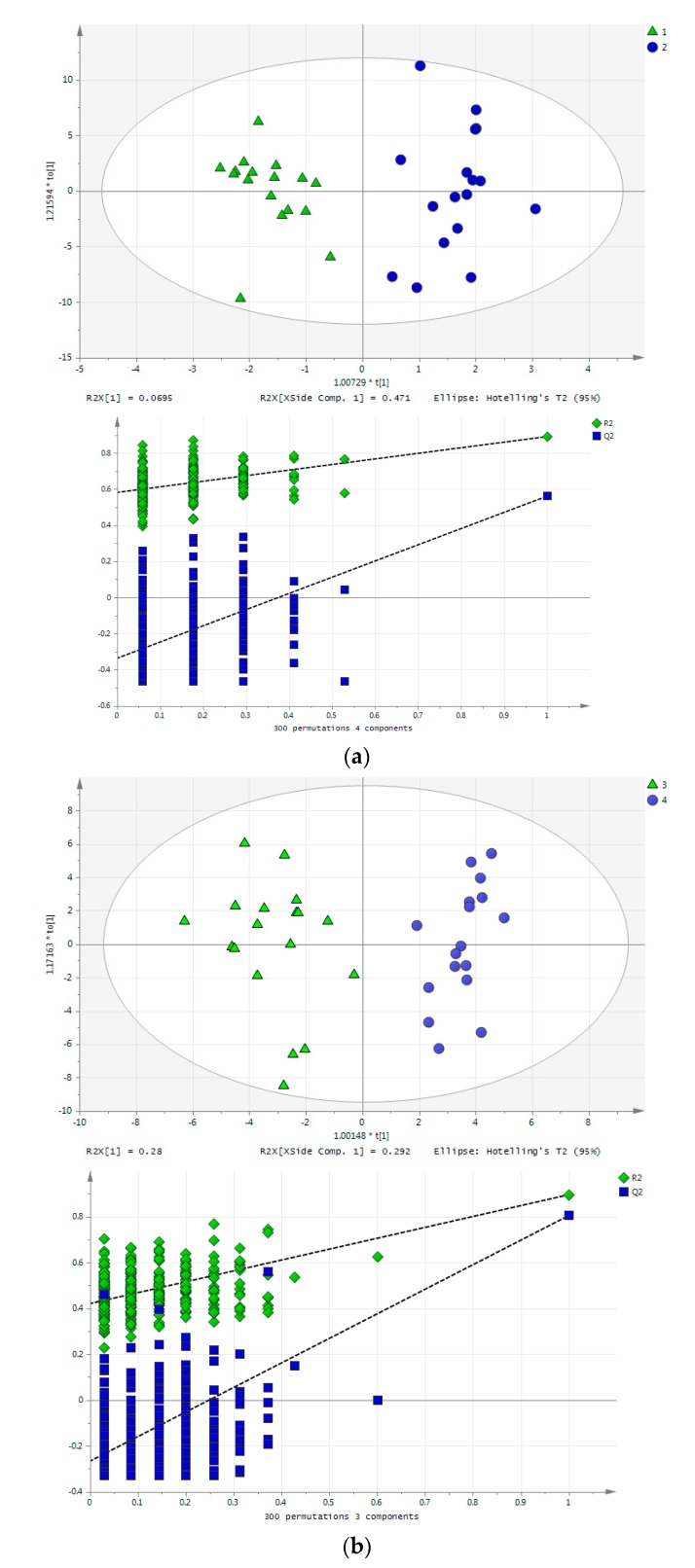
(**a**) OPLS-DA scores plot of rat whole blood samples of sedentary group A (blue) and exercise group B (green) at the first sampling time point. The permutation plot demonstrates a statistically significantly model; (**b**) OPLS-DA scores plot of rat whole blood samples of sedentary group A (blue) and exercise group B (green) at the second sampling time point. The permutation plot demonstrates a statistically significantly model.

In Figure 6a,b, the OPLS-DA scores plots of rat blood samples of life-long exercise group B and sedentary group A in both studied time points are presented. At the first sampling time point, corresponding to 3 months of rat exercise, 11 compounds (AUC values, 0.621–0.794) were found to be altered (alanine, aspartic acid, choline, glucose, glutamic acid, leucine, methylamine, proline, thymidine, trimethylamine, trimethylamine N-oxide) while at the second sampling time point, corresponding to 16 months of exercise, 19 metabolites (AUC values, 0.644–0.833) were altered (3-methylhistidine, acetylcarnitine, betaine, citric acid, citrulline, creatine, creatinine, cytosine, glucose, glutamic acid, glutamine, histidine, leucine, methylamine, niacinamide, ornithine, proline, pyruvic acid, tryptophan), based on both univariate and multivariate statistical approaches. Only five of them (glucose, glutamic acid, leucine, methylamine and proline) were common for both examined time points. 

Long-term exercise (for 16 months) affected blood metabolites at a greater scale. It is obvious that the training duration had a stronger influence than the intensity, as rats at the first sampling time point were trained with the maximum attached load. Changed metabolites were mainly associated with amino acids’, Krebs cycle’s, and pyruvate’s metabolism, which constitute hallmarks of the effect of exercise on the metabolome.

Rat blood samples were also examined in order to explore the impact of aging. Comparing either life-long exercise groups (Figure S6) or sedentary groups (Figure S7) between the two sampling time points, a clear effect of aging is observed. However, it can be seen that the separation is slightly inferior in the case of training rats which could be attributed to metabolic alterations due to exercise. Concerning the altered metabolites, 16 metabolites showed significant change in training rats and 23 metabolites showed significant change in sedentary rats. From these, 12 metabolites (aspartic acid, creatinine, cytidine, cytosine, glutamine, glycine, histidine, methionine, proline, thymidine, trimethylamine, trimethylamine N-oxide) were common in the two groups. Amino acids’ metabolic pathways, such as alanine, aspartate and glutamate’s pathway; glycine, serine and threonine’s pathway; arginine and proline’s pathway; cysteine and methionine’s pathway; and histidine’s pathway appeared to be related along with pyrimidine’s metabolism pathway. 

## 3. Experimental 

### 3.1. Samples

Urine and whole blood samples were collected from female Wistar rats, under the frame of an animal experiment performed in the animals’ facilities of Veterinary Medicine School of Aristotle University of Thessaloniki for a period of 17 months in accordance to the Helsinki Declaration and National standards (Permission code EL54BIO10). The rats’ initial population was 60, but there were small losses (three individuals) at the beginning of the experimentation. In the last month, the population was further diminished, due to physiological aging. Housing of the rats was under a regulated light/dark cycle of 12 h, in controlled temperature and humidity conditions. Female rats were preferred in accordance to international guidelines, enhancing female gender in experimental practices (US Department of Health & Human Services, NIH, Bethesda, MD, USA) and because aging related disorders such as frailty have been reported as being more evident in female mammals.

The study comprised three groups of animals, performing training for a short or a long period of time, and a control sedentary group. The training protocol was mild in order to simulate physical activity, since swimming is part of rats’ natural behavior [50].

Training included 15–18 min of swimming every day, 2–5 days per week, with a load of 4% to 0% of their body weight attached to rats’ tails.

Duration and intensity of training was adapted as the animals were aging from 5 months to 21 months, when they were sacrificed. Sedentary rats also came into contact with water, in order to avoid differentiations of the exercise rats due to the contact with the water. More details on the experimental protocol are provided in Deda et al., 2017 [43]. The groups of animals and the period of training are graphically described in Figure 1. Urine samples were collected at seven time points and blood samples were collected at two time points (shown in Figure 1). An additional set of urine samples was collected pre- and post-acute exercise session (the first day of the adopted protocol, after the mild acclimatization period of one week), (Figure 1). Urine samples were collected from the animals, strictly at a specific time of the day. All rats were fed ad libitum with standard chow and they were allowed to have free access to water. Body weight and food consumption were regularly monitored, and results were given in previous work (Supplementary Figure S1a,b, [43]). 

For urine collection, each rat was put onto a glass surface until urinating. The sample was directly collected by a pipette into eppendorf tubes and stored at −80 °C. For blood collection, rats were restrained into a transparent plexiglass tube specially prepared for this purpose and blood was collected from the lateral vein of the tail, using a very thin needle (23 G). This procedure is considered as mild and is well established in rat experimental procedures. Following bio-ethical guidelines, approximately 80 μL was collected from each rat in every collection sample time point. Directly after blood collection, a three-fold volume of methanol was added with high precision into the samples, in order to precipitate proteins. The mixtures were centrifuged for 10 min in 18,000× *g* at 4 °C and the clear supernatants were stored at −80 °C. Receiving this bio-fluid ensured that all samples were of the same quality, minimizing variations due to differences in hemolytic degree. 

### 3.2. Sample Preparation and Analysis

In the 20 μL urine sample, 60 μL of ice-cold acetonitrile was added; the mixture was centrifuged for 15 min (18,000× *g*) and the clear supernatants were inserted in LC-MS vials and immediately subjected to analysis. The protein-precipitated blood extracts were centrifuged for 25 min (18,000× *g*) after thawing and 60 μL of them was inserted in LC-MS vials for analysis. 

All samples were subjected to a targeted metabolomics analysis by an in-house HILIC-MS/MS method previously described in other publications of our group [43,51,52]. With the applied method, 113 metabolites are detectable with a high level of confidence in the metabolites’ identification. QC (Quality Control) samples were used in both rat urine and whole blood analysis to evaluate the precision of the analytic system [53,54,55,56].

### 3.3. Data Analysis

Data obtained from the LC-MS analysis were reprocessed using MassLynx^®^ (Waters, Milford, MA, USA) and TargetLynx^®^ (v4.1) software. The evaluation of the results was realized using Microsoft excel for univariate statistical analysis and SIMCA 13.0 (Umetrics, Malmö, Sweden) for multivariate statistical analysis based on peak areas data of the detected metabolites. Peak areas were not normalized and univariate scaling was applied for the construction of models. The validity of constructed models was evaluated using permutation plots (300 random permutations) and the value of CV ANOVA. Statistically significant compounds were also evaluated using ROC (Receiver Operating Characteristic) curve analysis. The AUC (area under the curve) of the ROC curve is given as an indicator of the accuracy of the separation of significant compounds. 

The online tools MetaboAnalyst 3.0 (Montréal, QC, Canada) [46], KEGG PATHWAY Database (Kyoto, Japan) [57] were used in order to investigate the related biochemical pathways and to illustrate their connection. 

## 4. Conclusions

The effect of exercise was demonstrated in the metabolome of rats’ urine and whole blood. The impact of exercise was observed at all examined sampling time points, but a higher impact of exercise was mainly observed as the length of the training period was increased. Long-term training could leave marks on urine metabolites, which may remain even after a period of 6 months, as has been shown in our results. Long-term exercise brings adaptations in related organs and can change glucose metabolism and enhance lipolysis even at rest, as Monleon et al. [28] concluded in their 18 months training study on a rat model. Based on our results, exercise altered amino acids-related pathways, purine’s degradation and acetylcarnitine which is a key metabolite in lipid oxidation.

In the acute session, exercise profoundly changed adenine, adenosine and glutamic acid, based on individual rat data evaluation. Huang et al. [25], who investigated rats in exhaustive and endurance exercise, concluded that there were alterations on amino acids, fatty acids, organic acids, and carbohydrates in rat liver metabolic profiles. Liver amino acids and Krebs cycle metabolites were also found to be altered in mice trained with maximal aerobic capacity [27].

Urine samples are considered to be a more suitable substrate in order to study exercise effect [31]. Our study proved that rats’ whole samples could be an equally reliable matrix for the investigation of exercise metabolic perturbations. Whole blood samples present the advantages that normalization could be avoided and also that the existence of high concentrated metabolites does not suppress the lower ones. Normalization is needed more in the analysis of urine samples, as differences in renal function and contained water could be reflected in the urine metabolic profiles [31]. In our studies, we avoid normalization, in order to keep pure biological responses. 

As far as the aging process is concerned, aging proved to be a stronger modifier in the analysed biofluid (urine and blood). The holistic changes that derived from the aging process influenced the majority of biochemical pathways reflected in rat metabolome. 

In both urine and whole blood samples, aging managed to change more metabolites than exercise. Further investigations are needed in order to decipher the changes in metabolites that are altered due to long-term exercising against aging.

## Figures and Tables

**Table 1:** Metabolites found to be responsible for the differentiation of rat urine samples, pre- and post-acute exercise session in both multivariate and univariate statistical approaches. VIP scores and *p*-values of each metabolite, sorted in descending VIP score order, are presented.

Significant Metabolites	VIP Score	*p*-Value
Thiamine	2.096	0.001
Adenosine	2.035	0.005
Putrescine	1.985	0.034
Adenine	1.950	0.005
Inosine	1.860	0.015
Acetylcarnitine	1.779	0.011
Niacinamide	1.723	0.016
3-(4-Hydroxyphenyl)lactate	1.572	0.045
Tryptamine	1.556	0.038
3-Methylhistidine	1.470	0.023
Glutamic acid	1.402	0.045
Creatine	1.384	0.048

## Supplementary Materials

The following are available online at www.mdpi.com/2218-1989/7/1/10/s1, Figure S1: OPLS-DA scores plot of rat urine samples of sedentary group A (blue) and exercise group B (green) at the fifth sampling time point. The inset permutation plot demonstrates a statistically significant model; Figure S2: OPLS-DA scores plot of rat urine samples of sedentary group A (blue) and exercise group B (green) at the seventh sampling time point. The inset permutation plot demonstrates a statistically significant model; Figure S3: PCA scores plot of rat urine samples before (blue) and after (green) the acute exercise session; Figure S4: PCA scores plot of life-long exercise rats (group B) of the first (blue colored) and last (green colored) sampling time point; Figure S5: PCA scores plot of life-long sedentary rats (group A) of the first (blue colored) and last (green colored) sampling time point; Table S1: Detected compounds found in analyzed urine samples using the applied HILIC-MS/MS method; Table S2: The effect of both aging and exercise (% fold change) on 10 metabolites affected by both exercise and aging; Table S3: Detected compounds found in analyzed whole blood samples using the applied HILIC-MS/MS method.
