# Supplementary material for: Impact of Exercise and Aging on Rat Urine and Blood Metabolome. An LC-MS Based Metabolomics Longitudinal Study
Source: Metabolites. 2017 Feb 23;7(1):10. doi: 10.3390/metabo7010010 (PMC5372213; doi:10.3390/metabo7010010)
Supplement: Supplementary file 1 [file metabolites-07-00010-s001.pdf]

Supplementary Materials: Impact of Exercise and Aging on Rat Urine and Blood Metabolome. An LC-MS Based Metabolomics Longitudinal Study

Olga Deda, Helen G. Gika, Ioannis Taitzoglou, Nikolaos Raikos and Georgios Theodoridis

Table S1: Detected compounds found in analyzed urine samples using the applied HILIC-MS/MS method.

Detected metabolites		
2-Hydroxy-3-methylbutyric acid	Guanine	Tryptophan
3-(4-Hydroxyphenyl)lactate	Histamine	Tyrosine
3-Methylhistidine	Hypotaurine	Myoinositol
Adenine	Hypoxanthine	N-Acetylaspartic acid
Adenosine	Inosine	Niacinamide
Alpha-Hydroxyisobutyric acid	Kynurenic acid	Ornithine
Alpha-Lactose	Acetylcarnitine	Pantothenic acid
Arabinose	Alanine	Putrescine
Betaine	Arginine	Pyridoxine
Biotin	Asparagine	Pyroglutamic acid
Cadaverine	Glutamic acid	Pyruvic acid
Choline	Glutamine	Raffinose
Citrulline	Histidine	Riboflavin
Cotinine	Isoleucine	Sarcosine
Creatine	Leucine	Sucrose
Creatinine	Lysine	Taurine
Cytidine	Malic acid	Thiamine
Arabitol	Methionine	Thymidine
Glucose	Phenylalanine	Trimethylamine
Dimethylamine	Proline	Trimethylamine N-oxide
Xylitol	Serine	Tryptamine
Xylose	Threonine	Uridine
Gamma-Aminobutyric acid	Mannitol	Valine
Glycine	Methylamine	

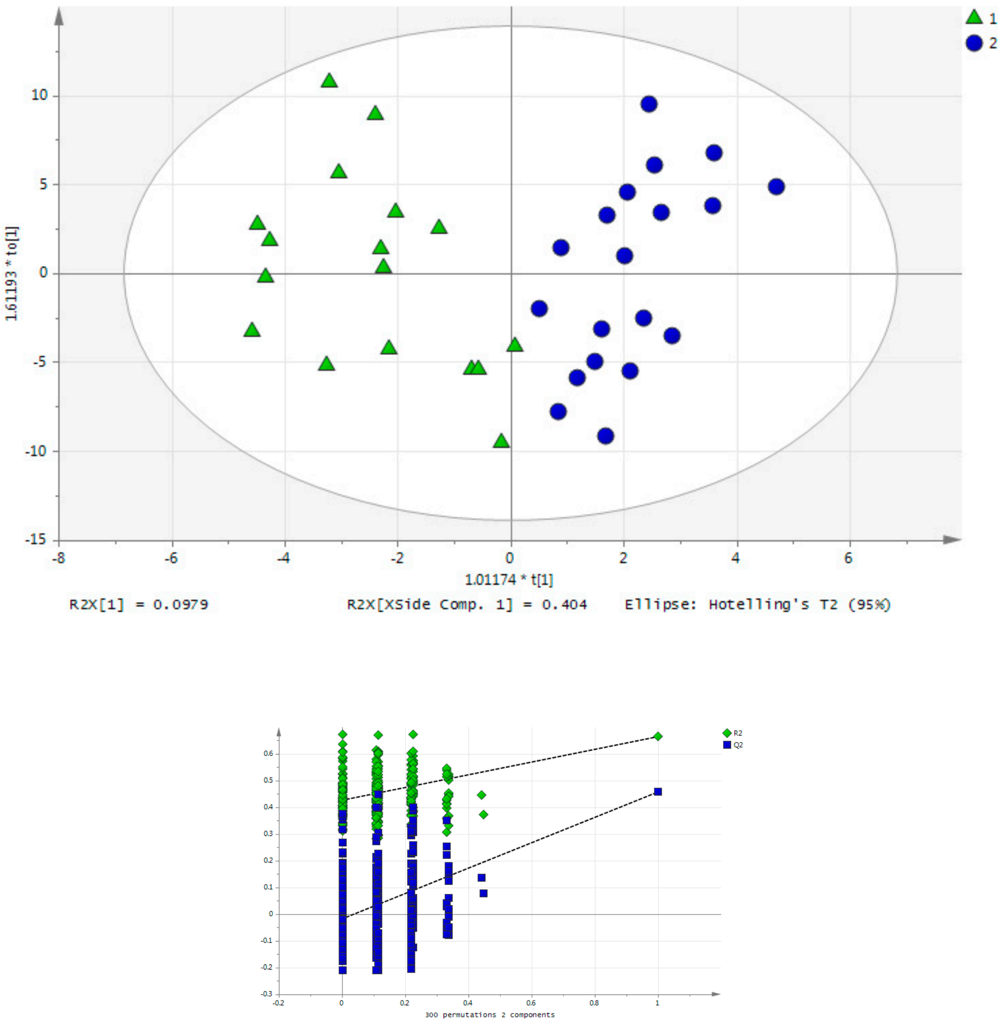

Figure S1: OPLS-DA scores plot of rat urine samples of sedentary group A (blue) and exercise group B (green) at the fifth sampling time point. The inset permutation plot demonstrates a statistically significant model.

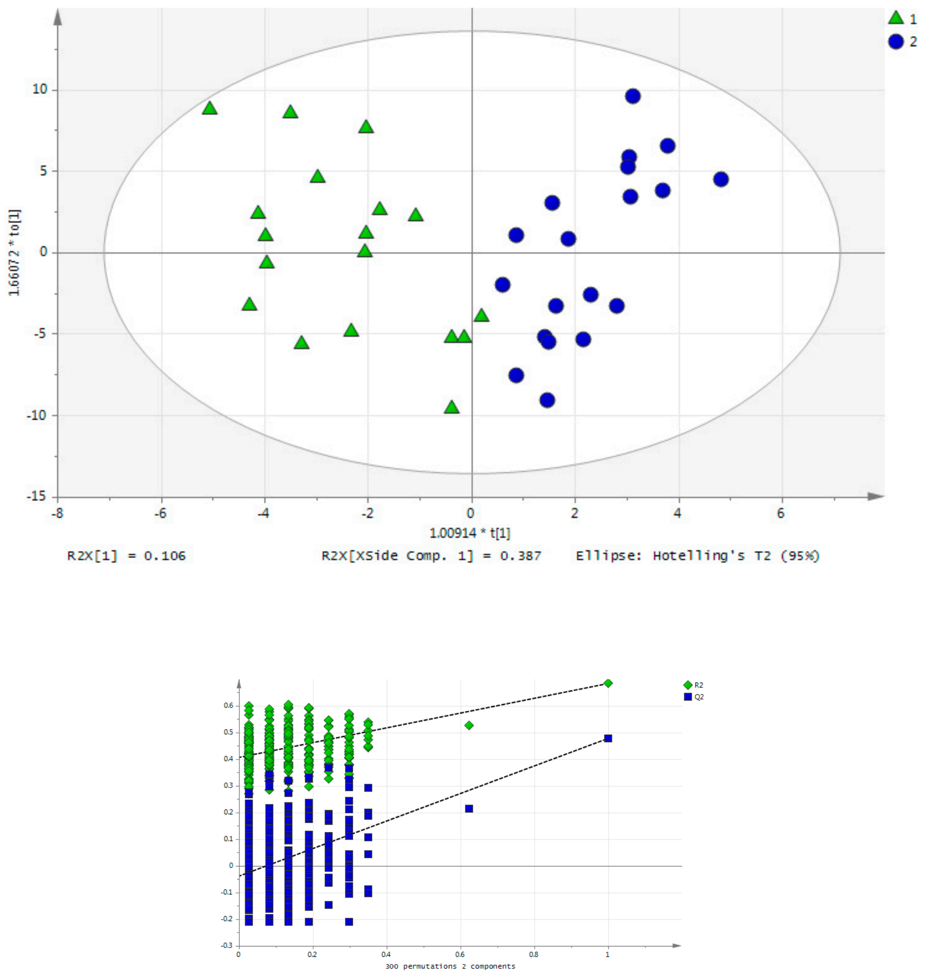

Figure S2: OPLS-DA scores plot of rat urine samples of sedentary group A (blue) and exercise group B (green) at the seventh sampling time point. The inset permutation plot demonstrates a statistically significant model.

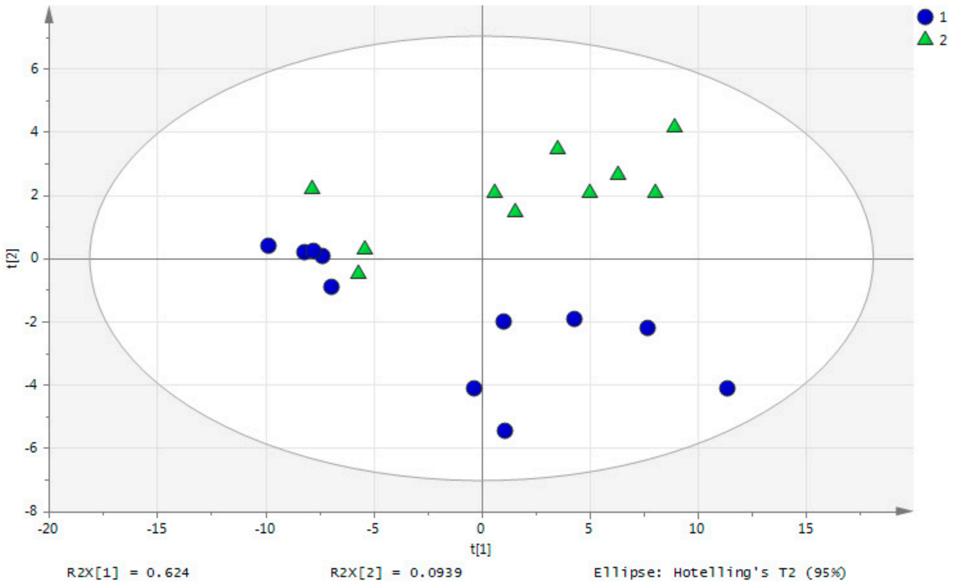

Figure S3: PCA scores plot of rat urine samples before (blue) and after (green) the acute exercise session.

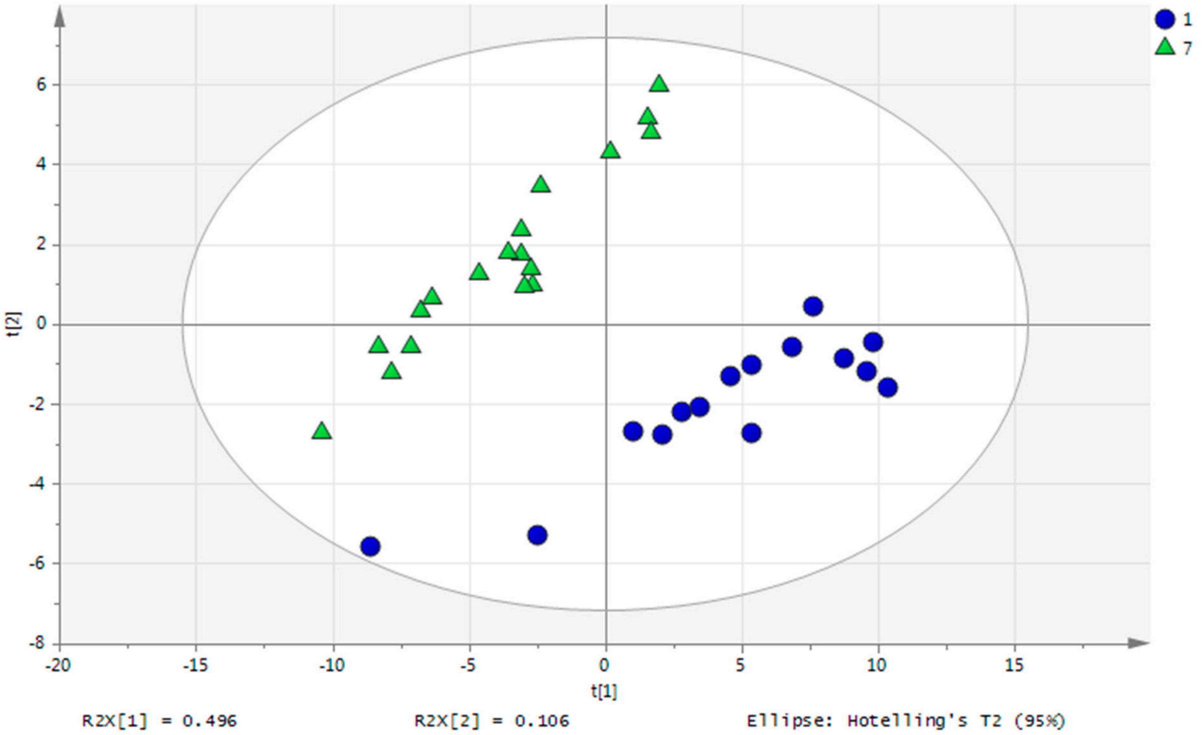

Figure S4: PCA scores plot of life-long exercise rats (group B) of the first (blue colored) and last (green colored) sampling time point.

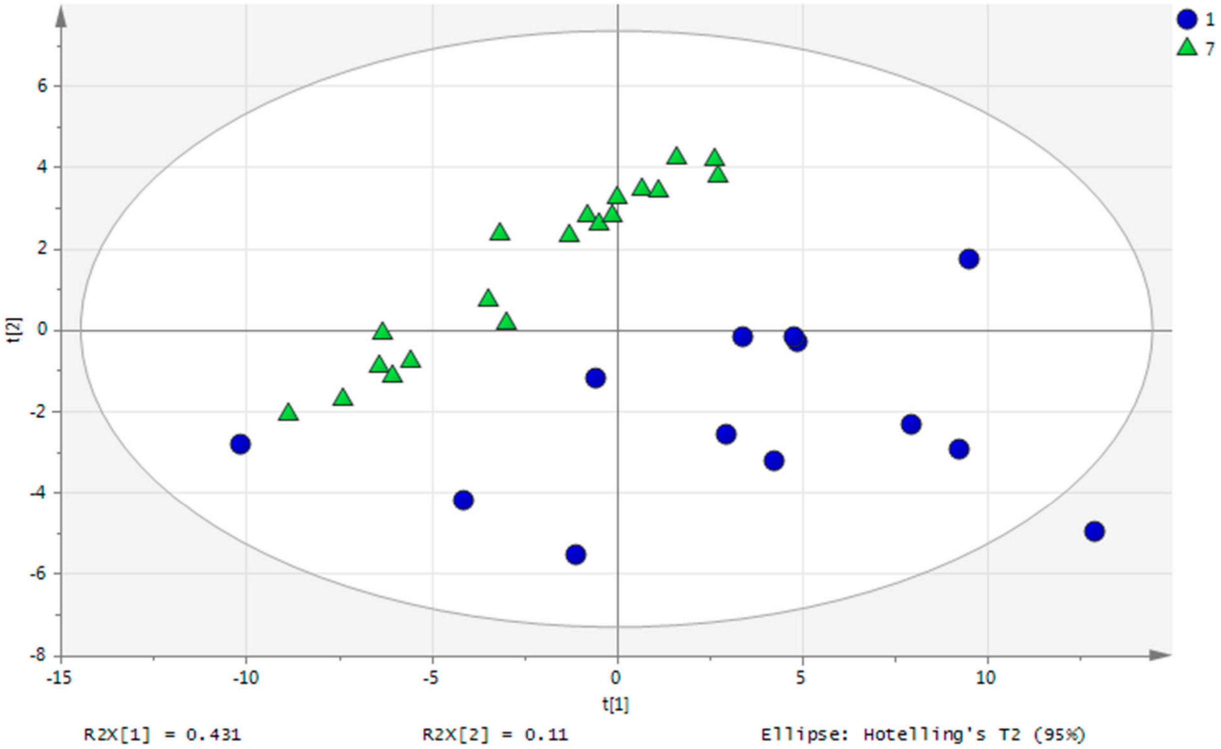

Figure S5: PCA scores plot of life-long sedentary rats (group A) of the first (blue colored) and last (green colored) sampling time point.

Table S2: The effect of both aging and exercise (% fold change) on 10 metabolites affected by both exercise and aging.

Metabolites	Aging	Exercise
Arabinose	-85	32
Methylamine	-73	32
Myoinositol	-58	41
Cotinine	-49	-6
Pantothenic acid	-39	14
Threonine	-23	24
Gamma-aminobutyric acid	-21	11
Leucine	-14	6
Creatinine	2	-1
Alpha-Hydroxyisobutyric acid	4	28

Table S3: Detected compounds found in analyzed whole blood samples using the applied HILIC-MS/MS method.

Detected metabolites	
3-Methylhistidine	Leucine
Adenine	Lysine
Adenosine	Methionine
Betaine	Phenylalanine
Choline	Proline
Citric acid	Serine
Citrulline	Threonine
Creatine	Tryptophan
Creatinine	Tyrosine
Cytidine	Methylamine
Cytosine	Niacinamide
Glucose	Ornithine
Glycine	Pantothenic acid
Histamine	Picolinic acid
Acetylcarnitine	Pyruvic acid
Alanine	Spermidine
Asparagine	Spermine
Aspartic acid	Thymidine
Glutamic acid	Trimethylamine
Glutamine	Trimethylamine N-oxide
Histidine	Uridine
Isoleucine	Valine
Lactic acid	

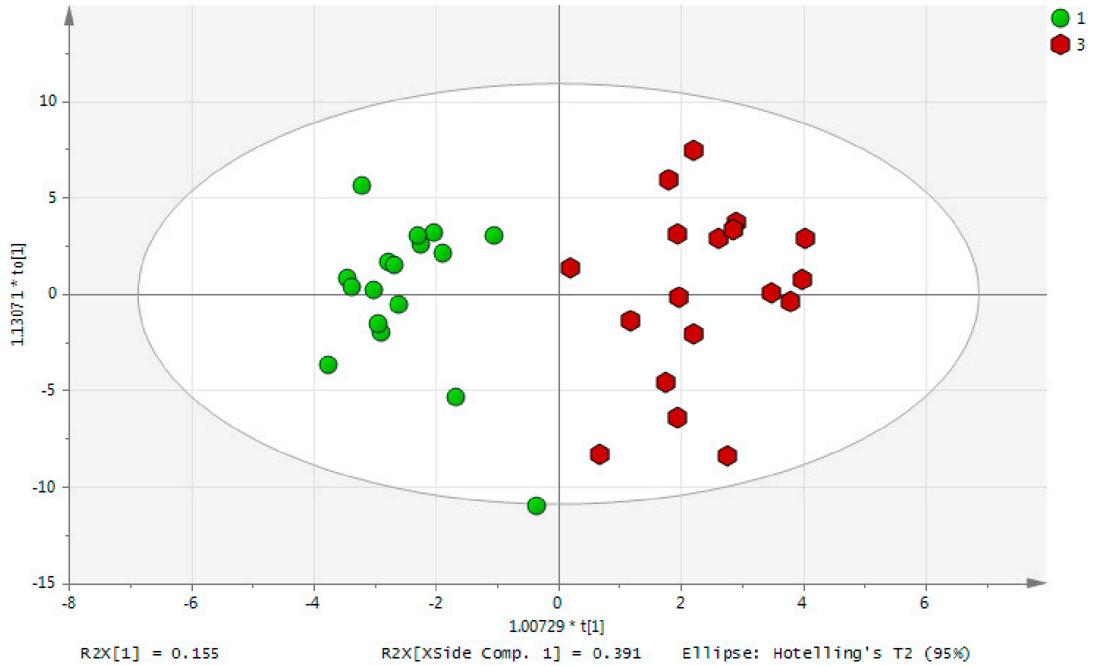

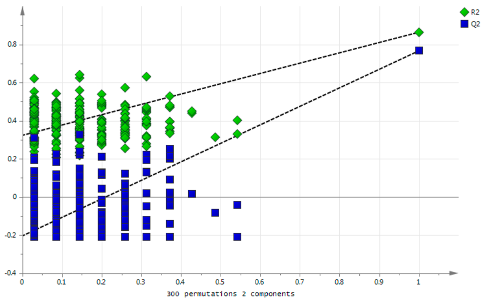

Figure S6: OPLS-DA scores plot of the whole blood exercise rat samples between the two sampling time points (8 months of age in green dots and 21 months of age in the red pentagon).

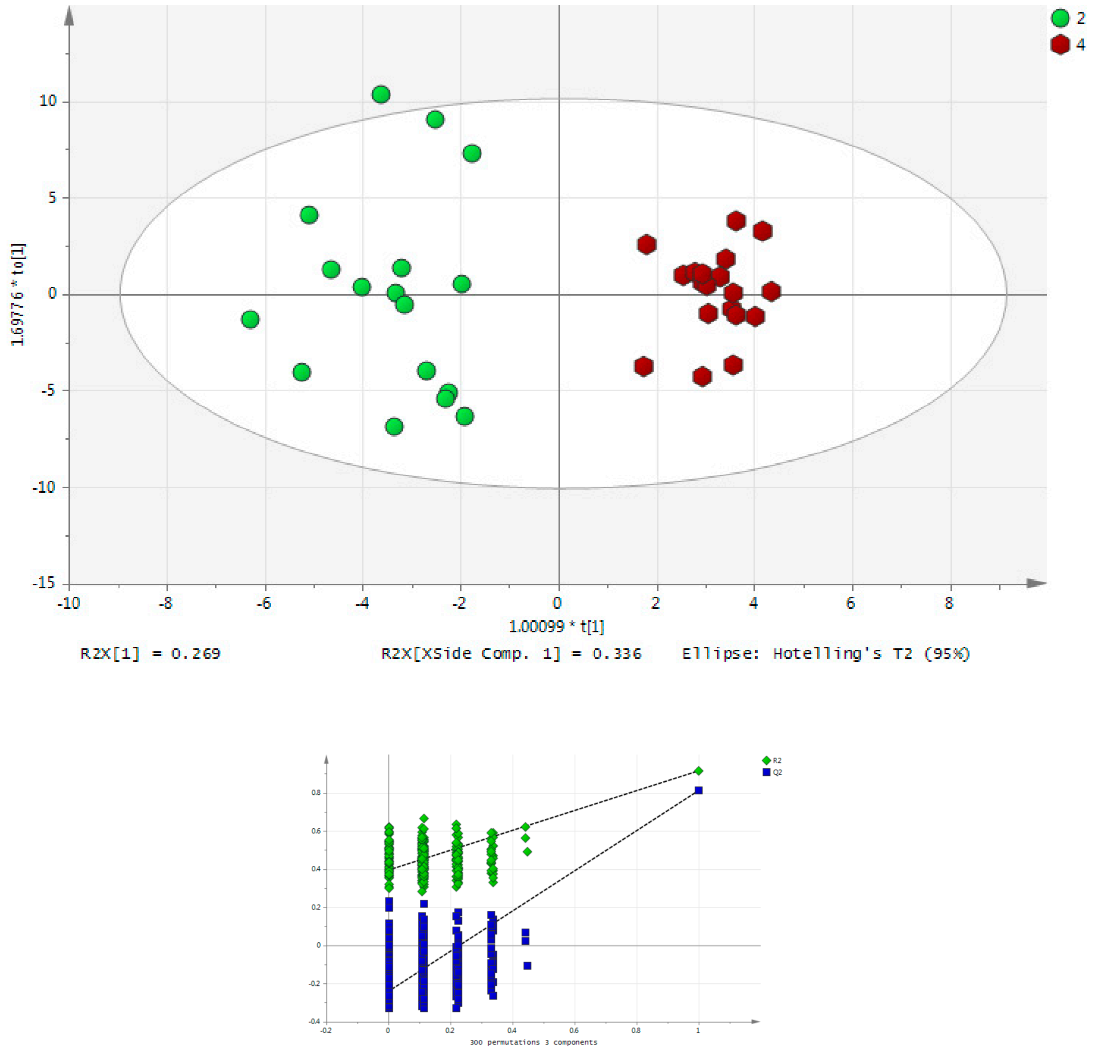

Figure S7: OPLS-DA scores plot of the whole blood sedentary rat samples between the two sampling time points (8 months of age in green dots and 21 months of age in the red pentagon).
